# Gaps in the knowledge and skills of Portuguese mothers associated with newborn health care

**DOI:** 10.1590/1518-8345.1859.2997

**Published:** 2018-05-07

**Authors:** Alexandrina Maria Ramos Cardoso, Heimar de Fátima Marín

**Affiliations:** 1 PhD, Associated Professor, Escola Superior de Enfermagem do Porto, Porto, Portugal.; 2 PhD, Full Professor, Universidade Federal de São Paulo, São Paulo, Brazil.

**Keywords:** Parenting, Mother-Child Relations, Child Health, Health Knowledge, Attitudes, Practice, Infant Welfare

## Abstract

**Objectives:**

assess mothers’ parenting knowledge and skills associated with the parental competence health promotion and monitoring for newborns and infants aged up to six months and determine the key characteristics of mothers who are better prepared for parenting.

**Method:**

cross-sectional study conducted in three health centers belonging to a Local Health Unit in the Northern Region of Portugal. Data was collected using clinical interviews conducted with pregnant women or mothers with a child aged up to six months. The tool used contained 21 child health promotion and monitoring indicators associated with different assessment moments: pregnancy, 1st/2nd week, 1st/2nd month, 3rd/4th month, and 5th/6th month.

**Results:**

we assessed the knowledge and skills of 629 women. Learning needs were identified for each of the indicators. The mothers who were better prepared for parenting tended to have a higher level of schooling, resided with the child’s father, had other children, had planned pregnancy, and intended to breastfeed.

**Conclusions:**

the results showed that knowledge and skills were lacking for each of the periods assessed by this study. First-time single mothers whose pregnancy was unplanned and who did not prepare themselves for parenthood may be considered a vulnerable group.

## Introduction

The mission of nursing is to facilitate transitions^1^. For both parents, having a child represents a developmental transition that requires the incorporation of new knowledge and skills to develop proficiency in child-care. 

Child health promotion and maintenance, specifically healthy growth and development, requires the active participation of informed and motivated parents who assume responsibility for caring for their children.

Preparation for parenting begins during pregnancy, when parents become aware of their role in ensuring their child achieves his or her maximum health potential. Parental behavior is therefore a key factor to ensuring healthy child development from the outset of pregnancy^2^ ^-^ ^5^. After birth, the child is totally dependent on parental responses to its behavior; thus, inadequate responses may negatively affect a child’s health. 

Good parenting is essential for ensuring a child’s survival, safety and well-being ^(^ ^6^. Therefore, developing parental competencies plays an important role in reducing neonatal morbidity^7^ ^-^ ^8^. 

Parental competence can be defined as a set of knowledge, skills and attitudes that enable effective parenting, thus ensuring that a child achieves maximum potential growth and development^9^ ^-^ ^10^. 

By mastering parenting skills, parents change the way in which they interpret their own performance and their child’s behavior. The higher the level of parenting knowledge and skills, the greater the likelihood of creating an environment conducive to healthy development and the greater the awareness of a child’s needs and the likelihood of responding to these needs^11^. 

By contrast, lack of parenting knowledge and skills may lead parents to underestimate their child’s capacity, meaning that the child is not stimulated to achieve his/her maximum development potential. Furthermore, unrealistic expectations in relation to parenting and child development can also increase the risk of abuse and neglect^8^ ^,^ ^11^ ^-^ ^12^. 

Given the lack of studies to sustain childbearing and parenting training programs focusing on health promotion and monitoring for newborns and infants aged up to six months, this study aimed to respond the following questions: what is the level of knowledge of mothers regarding the promotion and monitoring of their child’s health? What is the association between mothers’ knowledge and sociodemographic factors?

This study aimed to assess mothers’ parenting knowledge and skills associated with the parental competence health promotion and monitoring for newborns and infants aged up to six months and determine the key characteristics of mothers who are better prepared for parenting.

## Method

A cross-sectional study was conducted using data collected in three health centers in the Northern Region of Portugal after obtaining authorization from the Ethics Committee of the Local Health Unit. 

A total of 629 participants were selected using convenience sampling based on the following inclusion criteria: (1) being a mother - pregnant or with a child aged up to six months; and (2) being fluent in Portuguese. The mothers were initially approached by nurses working in the health centers and invited to participate in the study. All those who were approached agreed to participate. The study objectives and procedures were explained, confirming the voluntary nature of participation and the participants’ right to withdraw from the study at any time.

**Figure 1:**
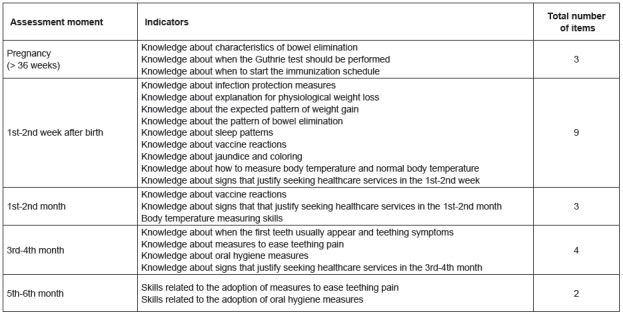
Parental competence for child health promotion and monitoring and respective indicators. Matosinhos, Portugal, 2010-2011(6)

Data was collected using a clinical interview conducted by the researcher. The interview guide included indicators of parental competence for child health promotion and monitoring contained in the Parental Competence Assessment Tool (*Instrumento de Avaliação das Competências Parentais* - IACP)^9^. The IACP was developed to systematize the assessment of parental competence during pregnancy and the first six months and consists of 193 indicators associated with 17 different competencies. It was designed based on the results of a content analysis of interviews conducted with 52 mothers, who were either pregnant or had children aged up to six months, and literature review. The tool was designed to provide a clinical assessment of mothers’ learning needs in order guide the diagnostic process and tailor nursing interventions to provide anticipatory care based on individual learning needs. The IACP contains 21 items associated with knowledge and skills needs related to child health promotion and monitoring that are important for decision-making/action-taking, 18 of which are specifically related to knowledge and three related to skills (Figure 1).

Making a clinical judgment based on the results of the interviews permits the formulation of a nursing diagnosis using dichotomous thinking. A clinical judgment “yes/present” permits to identify that the patient has adequate knowledge/skills. A clinical judgment “no” permits identify the identification of an opportunity to improve knowledge/skills^13^ ^-^ ^14^. 

The data collection tool was composed of two parts: the first addressing sociodemographic (age, level of schooling, marital status), obstetric (parity), and parental (intention to breastfeed, planned pregnancy, preparation for birth, preparation for parenting, sources of information) variables; and the second containing a set of 21 indicators associated with parental competence for child health promotion and monitoring. 

The internal consistency of the tool was measured using the Kuder-Richardson Formula 20 (KR-20), a special case of Cronbach’s Alpha for dichotomous variables (yes/no). Internal consistency was shown to be very good (KR-20 coefficient of 0.88; KR-21 coefficient of 0.87). 

Data analysis was performed using the IBM SPSS statistical software (version 19.0), using both descriptive and inferential statistics. Mothers’ knowledge and skills were scored between 0 and 1, based on the average score for the knowledge and skills items plus the “yes” answers (adequate knowledge/skill*)*, divided by the total number of items assessed. Items that were ‘not assessed’/‘not applicable’ were excluded from the equation to avoid bias. This resulted in the creation of six new variables: “total score”; “pregnancy score” (that included the three indicators assessed during pregnancy); “1st-2nd week score” (that included the nine indicators assessed during the 1st-2nd week); “1st-2nd month score” (that included the three indicators assessed during the 1st-2nd month); “3rd-4th month score” (that included the four indicators assessed during the 3rd-4th month); and “5th-6th month score” (that included the two indicators assessed during the 5th-6th month).

The exploratory analysis of skewness and kurtosis showed positive asymmetry skewed to the left with a leptokurtic distribution. Furthermore, the data had a nonnormal distribution (correction of the Kolmogorov-Smirnov test by Lilliefors). Nonparametric tests were therefore used, analysing the relationships between the scores and attribute variables using the Mann-Whitney and Kruskal-Wallis tests. When the Kruskal-Wallis tested indicated a significant difference (p < 0.05), the Mann-Whitney U test with Bonferroni correction (adequacy of p-value to test significance) was applied to identify in which group the difference was observed.

## Results

A total of 629 mothers particpated in the study. The average age of the sample was 29.5 years, 42% of the mothers had completed higher education, 32.7% had completed secondary education, and 25.3% had completed basic education. For 71.8% of the mothers it was their first child and 84.3% resided with the child’s father.

With respect to information sources (Table 1), during pregnancy, over half of the mothers sought advice from nurses, which rose to 75.8% in the 1st-2nd week after birth. The use of doctors as an information source also increased from 50.6% in the pre-natal period to 59.7% in the 1st-2nd week after birth. During these two periods, the least mentioned sources were family members and other mothers. 

**Table 1:** Information sources mentioned by the mothers (during pregnancy and in the 1st-2nd week after birth). Matosinhos, Portugal, 2010-2011

	Pre-natal (Pregnancy)	Post-natal (1st-2nd week)
Nurse	51.1%	75.8%
Doctor	50.6%	59.7%
Books	55.0%	38.6%
Internet	50.8%	37.3%
Family members	27.6%	23.3%
Other mothers	19.9%	9.3%

The results show that the mothers lacked knowledge and skills for all items. Over 50% of mothers were shown to lack overall Knowledge about the items related to pregnancy, 65% did not know when the screening test for the early detection of congenital disorders (the Guthrie test) should be performed, 62% did not know when to start the immunization schedule, and 55% were unaware of the characteristics of bowel elimination. 

With respect to the 1st-2nd week, 65% of mothers lacked Knowledge about the expected pattern of weight gain, 64% regarding signs that justify seeking healthcare services, and 62% regarding sleeping patterns. Furthermore, over half of the mothers lacked Knowledge about physiological weight loss (53%) and how to measure body temperature (58%). Almost half of the mothers lacked Knowledge about jaundice and normal coloring (49% and 47%, respectively). A little over 40% of mothers lacked Knowledge about vaccine reactions (43%) and infection protection measures (41%), while 31% showed a lack of knowledge in relation to the pattern of bowel elimination. 

With regard to the indicators assessed during the 1st-2nd month, half of the mothers lacked Knowledge about signs that that justify seeking healthcare services and vaccine reactions (51% and 49%, respectively), while 39% lacked body temperature measuring skills. 

With respect to the 3rd-4th month, a significant amount of mothers (86%) showed lack of Knowledge about oral hygiene measures. Furthermore, 64% of mothers lacked Knowledge about measures to ease teething pain, 53% we unaware of the signs that justify seeking healthcare services in this phase of development, and 39% were unable to recognize teething symptoms. 

With regard to the 5th-6th month, 64% and 40% of mothers needed to improve their skills related to the adoption of oral hygiene measures and measures to ease teething pain, respectively.

Table 2 below shows that the average knowledge and skills scores for each assessment moment (Table 2), revealing a gradual decrease for consecutive periods.

**Table 2:** Average knowledge and skills scores for each assessment moment. Matosinhos, Portugal, 2010-2011

Scores (items)	Average (SD)
Pregnancy score (3 items)	0.358 (0.38)
1st-2nd week Score (9 items)	0.157 (0.27)
1st-2nd month Score (3 items)	0.130 (0.30)
3rd-4th month Score (4 items)	0.085 (0.22)
5th-6th month Score (2 items)	0.084 (0.24)

The highest score was obtained for the assessment moment pregnancy (A = 0.36; DP = 0.38), while the lowest scores were obtained for the 3rd-4th month and 5th-6th month (0.085 and 0.084, respectively). 

**Figure 2:**
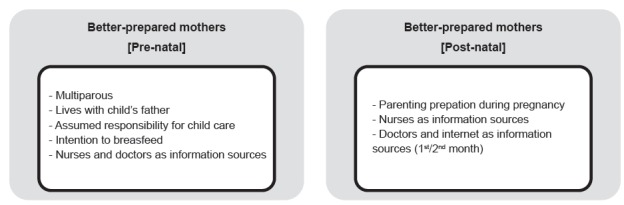
Summary of the key characteristics of better-prepared mothers in terms of parental competence for child health promotion and monitoring. Matosinhos, Portugal. 2010-2011

To determine the key characteristics of mothers who are better prepared for parenting, we analyzed the association between level of knowledge and skills and the attribute variables (Figure 2). 

During pregnancy, it was shown that mothers who had other children (U = 28465; p < 0.001), who resided with the child’s father (U=19735; p<0.001), who assumed responsibility to care for their newborn child themselves (U=8859; p<0.001), who intended to breastfeed (U=25517; p<0.001), and who sought advice from nurses and doctors as information sources during pregnancy (U=41176; p<0.001 and U=42520; p=0.004, respectively), tended to have more knowledge. 

In the post-natal period, it was shown that mothers who had attended parenting preparation classes during pregnancy, who intended to breastfeed and consulted a nurse in 1st-2nd week, 3rd-4th month, and 5th-6th month (U=3488, p=0.001; U=3999, p=0.011; U=3332, p=0.045, respectively), and who consulted a doctor and the internet as information sources in the 1st-2nd month (U=4903; p=0.010 and U=4850; p=0.015, respectively), tended to have more knowledge. 

## Discussion

Mother’s are mainly responsible for child care^(9.15)^ and therefore the quality of care is influenced by the mother’s knowledge and skills regarding the health of her child.

The findings show that women who had had more than one child and resided with the child’s father tended to more knowledge and skills needed child health promotion and monitoring. These findings are in line with the results of other studies that show that first-time mothers are more likely to have poor Knowledge about child development ^(^ ^7^ ^-^ ^8^. Another study reported that there was no association between mothers’ knowledge of child health and level of schooling and number of children^15^. In contrast, a different study showed that mothers who had a low level of schooling were more likely to lack Knowledge about jaundice in newborns ^(^ ^16^. 

The present study did not observe an association between mothers’ knowledge and skills and age and schooling level. However, other studies have shown an association between level of knowledge of Guthrie test in the neonatal period and level of schooling ^17^. Other authors reported that an association between schooling level and scores for the items recognition of warning signs, treating the umbilical stump and measuring and interpreting body temperature^8^. A study conducted in 2005 reported that schooling level was a strong predictor of mothers’ knowledge^5^. 

Nurses and doctors were the most commonly mentioned information sources and were these sources that had the greatest influence on the knowledge and skills of mothers, both in the pre-natal and post-natal periods. This finding is consistent with the results of other studies conducted in Europe, with a study undertaken in Italy showing that 42% of mothers consulted pediatricians as the main source of information about vaccines(18). Another study conducted in a country in the Middle East reported that the main source of information (80%) was family members (grandmothers, mothers, sisters, etc.) and that a mere 7.1% of mothers consulted health professionals (15). These findings show that cultural factors may influence the types of sources used by mothers to gain information about childcare. 

### Knowledge about the Guthrie test

The results of the present study show that 60% of mothers lacked basic Knowledge about child health during pregnancy, particularly regarding the Guthrie test and immunization. A study conducted in Brazil to assess mothers’ Knowledge about the Guthrie test among 110 women who had recently given birth, observed that 97% of the women had heard of the test, but were unaware of the purpose and importance of the test ^(^ ^19^. 

### Knowledge about immunization

Besides not knowing when to start the immunization schedule, over 40% of the mothers interviewed by the present study lacked knowledge of common vaccine reactions in babies. This finding is similar to those of other studies. A study conducted to assess the knowledge of pregnant women in Brazil (N=65) about child health showed that only 29.3% knew when to start vaccination and that none of the mothers knew which vaccines should be administered^20^. Another study with 223 mothers observed that although a large proportion of the mothers were aware of common reactions to the BCG vaccination, only a little over half (51.3%) knew that it is administered to protect children from tuberculosis^7^. A further study that interviewed 30 mothers (15 with one child and 15 with more than one child) to assess vaccination information needs and information seeking behavior showed that only four women (two with one child and two with more than one child) knew the name and purpose of the vaccine administered to their children ^(^ ^3^. Another study conducted in Italy observed that only 26% of the participating mothers knew which vaccines made up the vaccination schedule^18^.

### Knowledge about warning signs

Half of the mothers interviewed under the present study lacked the necessary knowledge to recognize warning signs and know when to seek healthcare services. Another study observed that mothers had a satisfactory level of knowledge of warning signs in newborns and infants in the first few months of life^7^.

Recognizing warning signs can be particularly complex given the broad spectrum of normality patterns in child health. A study conducted with 373 mothers to assess their knowledge of specific aspects of child health showed that half of the mothers were not aware that a newborn that “only sleeps and does not feed” may be ill and need urgent attention and that 25% of mothers did not know that the fact that a child “cries continually, doesn’t sleep and expresses pained when moved” is a warning sign and that the child requires immediate attention^15^. 

The present study also showed that almost half of the mothers (49%) lacked knowledge about jaundice. Similar results were obtained by other studies. A study conducted with 161 mothers to assess their knowledge about neonatal jaundice showed that 53.6% of the mothers had inadequate knowledge^16^, while another study undertaken with 396 mothers who had recently given birth to determine their knowledge, attitude and behavior in relation to neonatal jaundice showed that 53% (N=203) had little knowledge about this problem ^(^ ^21^. Another study highlighted that it is was worrying to find that the majority of mothers thought that jaundice in the first Day of life of a newborn is normal and simply required more frequent feeding and exposure to sunlight^15^. 

### Knowledge about body temperature

A considerable number of mothers lacked knowledge (58% in the 1st-2^nd^ week) and skills (39% in the 1st-2nd month) for measuring and interpreting body temperature. This finding is consistent with the results of a literature review addressing the knowledge, attitudes and practices of parents in relation to fever in children, which revealed that mothers’ knowledge of what is considered normal body temperature and what is considered a fever was poor^22^. Another study showed that 35.1% of mothers thought that fever was an indication of a serious disease ^(^ ^15^ ^).^

### Knowledge about newborn weight

Weight loss or low weight gain in newborns is source of doubt for most mothers, particularly those who breastfeed. Our findings show that over half of the mothers lacked knowledge about the expected pattern of weight gain (65%) and physiological weight loss (50%). Given that in exclusively breastfed babies milk intake is difficult to ascertain, weight is the best indicator of nutritional status in the first year of life^23^. 

The results also show that over 60% of mothers did not have any knowledge about child sleeping patterns. Similar results were found by another study involving 203 mothers, which showed that child sleeping patterns was the area in which the participants most lacked knowledge^5^. 

### Knowledge about oral health

The mothers also showed a lack of knowledge and skills in relation to childe oral health. Oral hygiene routine should be a routine component of parenting behavior, even before the first teething episode(20). A study that assessed the knowledge of Brazilian mothers of children aged between zero and four years in relation to child oral health promotion showed that only 21% of mothers initiated oral hygiene before the first teething episode and that cloth diapers and damp gauze were the most commonly used items for performing oral hygiene(59%)(24). 

Over half of the mothers obtained low scores (weak and reasonable) for this set of knowledge and skills, showing that there was room for improvement. 

Another study, using a tool with a maximum score of 40 and a cut-off score of 25, observed that 58.4% of mothers had a satisfactory level of knowledge about child health. However, the researchers highlighted that if the cut-off score had been 30, only 12% of mothers would have shown a satisfactory level^15^. A further study observed that 65% of mothers correctly answered all questions on the questionnaire regarding child development^5^. 

The mothers that participated in the present study showed greater knowledge in relation to the items associated with pregnancy, showing a gradual decrease in average scores for consecutive moments of assessment. This finding could be explained by the fact that the search for information and likelihood of contact with health professionals is generally greater during pregnancy than in later phases of child development. This idea is somewhat confirmed by other authors when they observe that after birth mothers and children “compete” for the attention of health professionals^25^. 

This study showed some limitations in relation to sampling, given that convenience sampling does not allow for the extrapolation of results to other population groups. Another limitation relates to the definition of assessment criteria. Although the assessment criteria used for gauging knowledge and skills were validated by experts in the field of knowledge in question (academics and nurses), the systematization of nursing diagnosis is an area that requires further research. 

## Conclusion

This study revealed gaps in the knowledge and skills of mothers in relation to health promotion and monitoring for newborns and infants aged up to six months. 

The results highlight the areas in which knowledge and skills are most lacking. With respect to the items associated with pregnancy, the mothers showed a lack of knowledge about the Guthrie test and immunization, while in the 1st-2nd week knowledge was poorest in relation to weight gain, warning signs and sleeping patterns. With regard to the 1st-2nd month, warning signs stood out as the area in which the highest percentage of mothers lacked knowledge. With respect to the 3rd-4th month, lack of knowledge was greatest in relation to oral hygiene and how to ease teething pain, while in the 5th-6th month the majority of mothers lacked skills related to the adoption of oral hygiene measures. 

The mothers who were better-prepared for child health promotion and monitoring tended to have a higher level of schooling, had other children, resided with the father of the child, and consulted nurses as information source after the birth of their child. In contrast, first-time single mothers whose pregnancy was unplanned and who did not prepare themselves for parenthood may be considered vulnerable.

### Practical implications

Nurses play a critically important role in the development of parenting skills. By identifying the main gaps in parenting knowledge and skills, the findings of this study can inform the design of parenting training programs focusing on health promotion and monitoring for newborns and infants aged up to six months. Indeed, the systematic assessment of the learning needs identified in this study could constitute a starting point for defining the content of nursing interventions contemplated by the nursing care plan
